# Fractional Coprecipitation of Drugs and Natural Extracts with Zinc Hydroxide

**DOI:** 10.3390/molecules30132699

**Published:** 2025-06-23

**Authors:** Andrea Franzese, Luca Regazzoni

**Affiliations:** Department of Pharmaceutical Sciences, University of Milan, Via Mangiagalli 25, 20133 Milan, Italy

**Keywords:** zinc hydroxide, precipitation, fractionation, drugs, natural extracts

## Abstract

Zinc hydroxide has been reported as an effective precipitating reagent for removing proteins in biological samples. This procedure is quite effective for removing interfering proteins before the chromatographic separation of small organic compounds. However, preliminary data suggested that also some small molecules could precipitate together with proteins and zinc hydroxide. Therefore, herein it is reported a study on a panel of drugs having different chemical structures. The results suggest that the common trait of organic molecules coprecipitating with zinc hydroxide is to have acidic groups, while neutral or basic molecules are not affected by zinc hydroxide precipitation. Such observations were consistent with some analyses conducted on hydroalcoholic extracts prepared from natural edible materials such as green tea. In such matrices, a quantitative coprecipitation of polyphenols was obtained upon inducing the precipitation of zinc hydroxide, while alkaloids such as caffeine remained selectively isolated in the supernatants. Interestingly, the compounds coprecipitated with zinc hydroxide can be easily and quantitatively recovered as well, just by redissolving the precipitate. These findings open potential applications for the isolation of specific classes of compounds from crude natural extracts and for the use of zinc hydroxide to remove interfering compounds before chromatographic analyses.

## 1. Introduction

Fractional precipitation is a common method to separate compounds having different solubilities. Such a procedure is used for a wide array of applications in different fields. For instance, the selective precipitation of metals through the formation of insoluble salts or complexes has been reported for environmental remediation [1,2,3] or for metal recycling from waste materials [4,5,6].

Concerning applications targeted to organic molecules, fractional precipitation is especially used for the purification or the fractionation of polymers, since molecules having different sizes typically have also a different solubility. Few examples reported in literature are the fractionation of cellulose triacetate [7], poly (buta-diene-co-styrene) [8], and carrageenans [9]. Chemical reactions like those used for metal precipitation are not the only way to operate fractional precipitation. Temperature can be also manipulated to induce fractionation as demonstrated for polyethylene oxide polymers [10].

The fractional precipitation is extensively used also for the analysis and purification of biomolecules. The purification of proteins is the application with the most extensive literature [11]. Over the years many studies have been reported to test the effects of different chemical reagents on protein solubility. Such studies were aimed at optimizing efficient, cheap, scalable, and consistent methods allowing the isolation of selected proteins through a controlled precipitation. The most popular methods are based on the addition of chemicals to modify polarity, ionic strength or pH of the solutions thereby triggering protein precipitation [12,13]. Ammonium sulfate is quite popular when protein precipitation is intended for protein isolation. On the contrary, organic solvents and acidic reagents are less popular because they can cause alterations in protein structure and protein recovery from the precipitate is easier with protocols based on ammonium sulfate [11,13,14]. Sometimes the protocols can include the combination of different chemicals to achieve the desired degree of purification, as in the case of the fractionation of plasma proteins according to Cohn protocol [15]. Heat can also be used for inducing protein denaturation and precipitation, but thermal shocks often cause an irreversible modification of protein structure and for this reason they are not the most popular choice for protein purification [16].

Alternatively, fractional precipitation is also popular to permanently remove proteins from the samples, especially before chromatographic separations or other analytical procedures intended for the identification or quantitation of small molecules. Organic solvents (e.g., acetonitrile, acetone) and acids (e.g., perchloric acid, trichloroacetic acid) are the most popular choices since the analyses are targeted at small, soluble molecules and the irreversible denaturation of precipitated proteins is not an issue. The literature reports many comparative studies describing the advantages of the use of different reagents to remove interfering proteins through selective precipitation [17,18,19,20].

Recently, a study was published on the optimization of a protein removal method based on the addition of zinc salts and basic compounds into the sample to trigger zinc hydroxide precipitation [21]. Such procedure determine the coprecipitation of proteins with zinc hydroxide and was successfully tested to remove interfering proteins before chromatographic separations aimed at the quantitation of small molecules in biological matrices [21]. Such a procedure has some apparent advantages over other protocols based on the use of organic solvents (e.g., acetonitrile and methanol) or acidic reagents (e.g., trichloroacetic acid and perchloric acid). In fact, protein precipitation by zinc hydroxide can be achieved without altering sample pH or polarity [21]. The result obtained by analyzing small molecules in plasma suggest that analyte recovery can be quite variable. In fact, while lidocaine concentration was not changed in the supernatant upon zinc hydroxide precipitation, for the analyte 3 hydroxy anthranilic acid a 40% reduction of concentration was instead observed. Since other precipitating reagents can also cause low analyte recovery such data can fall within an acceptable range for the validation of analytical methods [20,22]. However, such evidence suggests that small molecules can possibly coprecipitate with zinc hydroxide. This hypothesis is reinforced by some literature data reporting a low recovery for aminoacids when samples were spiked with zinc sulfate first, followed by barium hydroxide [17]. The study was performed to find the optimal protocol for the analysis of free aminoacids in plasma and the use of zinc sulfate and sodium hydroxide was adopted based on protocols reported in literature. However, recent mechanistic insights evidenced that the combination of zinc sulfate and barium hydroxide trigger the precipitation of zinc hydroxide [21]. Therefore, the coprecipitation of aminoacids with zinc hydroxide is the most likely explanation of the low recovery observed. Such a hypothesis is also sustained by other pieces of evidence based on experiments performed on enzymes [23]. On the contrary there is no consistent evidence that the compounds used for the precipitation of proteins can be of any use for the selective fractionation of mixtures of small organic compounds. In fact, organic solvents and acidic reagents like trichloroacetic acid and perchloric acid can remove proteins without affecting the solubility of wide array of organic compounds [17,18,19,20].

Therefore, herein it is reported a study aimed at better understanding whether zinc hydroxide coprecipitate also small organic molecules with structures different from aminoacids or proteins. The study was designed to find whether zinc hydroxide preferentially interacts with some specific class of compounds, or with molecules having specific structural features. Moreover, the protocol was also tested for potential applications for the fractionation of mixtures of small organic molecules. This represented a new application of fractional precipitation, since such a technique is more popular for the isolation, removal or fractionation of polymers and macromolecules of biological origins. On the contrary the data herein reported support the hypothesis that zinc hydroxide can be used for the fractionation of mixtures composed of small molecular weight molecules of synthetic and natural origins.

## 2. Results and Discussion

### 2.1. Fractional Coprecipitation of Drugs with Zinc Hydroxide

**Figure 1:**
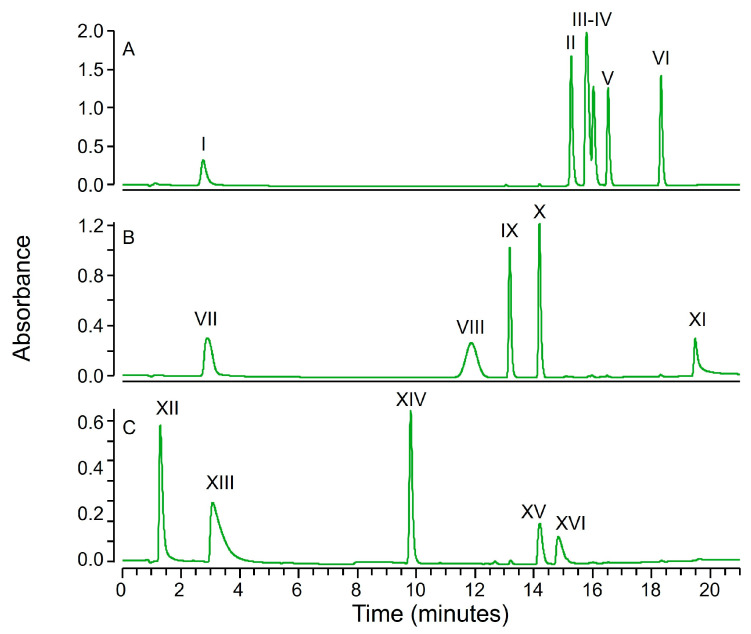
LC-UV chromatograms (220–400 nm) of mixture (**A**) containing Acetazolamide (I); Piroxicam (II); Telmisartan (III); Losartan (IV); Ketoprofen (V) and Diclofenac (VI); mixture (**B**) containing Acetaminophen (VII); Edaravone (VIII); Phenacetin (IX); Prednisolone (X) and Clioquinol (XI); and mixture (**C**) containing Hydralazine (XII); Procaine (XIII); Caffeine (XIV); Bromhexine (XV) and Fluoxetine (XVI).

The tests were performed on a panel of 16 drugs. As reported in Figure 1, the compounds were tested as mixtures and the effect of zinc hydroxide precipitation on single analytes was monitored after chromatographic separation using both an UV and an MS detector (see Section 3.4).

**Figure 2:**
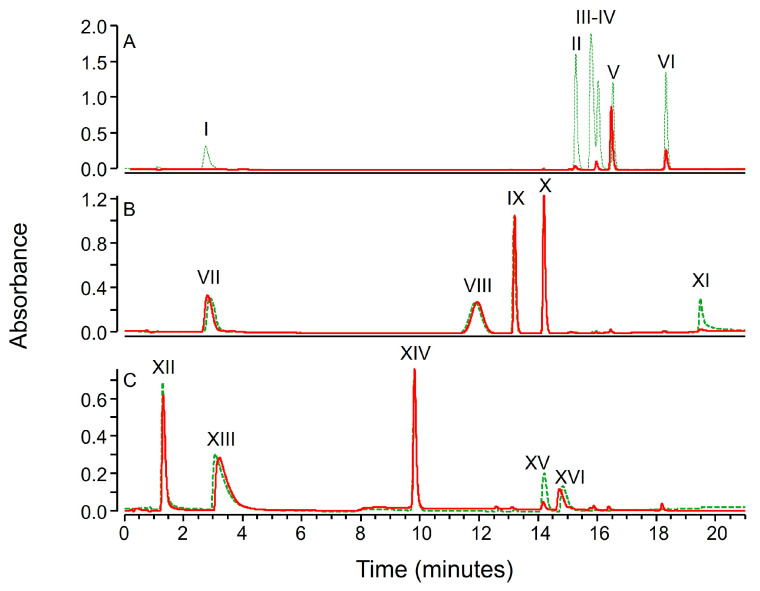
LC-UV chromatograms (220–400 nm) of mixtures (**A**–**C**) (green dashed line) and of the supernatants of mixtures (**A**–**C**) obtained after inducing the precipitation of 5 mM zinc hydroxide (solid red line).

As recently reported, zinc hydroxide precipitation was triggered into the samples by the addition of zinc chloride first, and sodium hydroxide next [21]. The analysis of the supernatants obtained upon the removal of zinc hydroxide precipitate revealed different behaviors for the compounds. Specifically, the chromatograms of the mixtures B and C were almost unaltered since only the peak area of compounds XI and XV was reduced by zinc hydroxide precipitation (see Figure 2B,C). Therefore, most of the compounds contained in mixture B and C seem not to coprecipitate with zinc hydroxide. On the contrary, nearly all the compounds contained into mixture A were undetectable or barely detectable upon zinc hydroxide precipitation (see Figure 2A). This suggests that zinc hydroxide can be quite effective as precipitating reagents also for some small molecules, with some degree of selectivity.

Data were reproducible across samples since the analysis of three independent samples gave similar results for all compounds in term of the residual amount detectable in the supernatants. The average relative standard deviation for the entire dataset (i.e., residue of all 16 compounds included, UV and MS data combined) was 6.1%. Only data collected for compounds VII and XII have a relative standard deviation above 10%. This higher relative standard deviation derived from a sample where the estimated residual of compounds VII after zinc hydroxide precipitation was 137% of the initial amount (by MS), and another sample where the estimated residual of compounds XIII after zinc hydroxide precipitation was 124% of the initial amount (by UV). Both data were considered as not relevant since they still indicate that such compounds do not coprecipitate.

Such findings are interesting since the use of zinc hydroxide was recently demonstrated as a useful method for fractional precipitation of proteins [21]. On the contrary, no application for fractional precipitation of small molecules has yet been reported. The only evidence of potential effects of zinc hydroxide on the precipitation of small molecules is a paper reporting a study on the performance of several protein precipitating reagents. The paper reports a low recovery of aminoacids upon treatment with zinc sulfate and barium hydroxide [17]. Nevertheless, while aminoacids share structural similarities with proteins, the data reported in Figure 2 have been obtained with drugs having structures that are quite different from an aminoacidic or peptidic scaffold. This suggests that the mechanism of coprecipitation is not selective towards aminoacids, peptides or proteins: Therefore, it can be speculated that some structural features should determine the coprecipitation with zinc hydroxide. To detect which structural features are typical of molecules coprecipitating with zinc hydroxide an unsupervised multivariate analysis was performed on a dataset including for each compound the residual percentage measured in the supernatant after zinc hydroxide precipitation (average of three independent samples measured both by LC-UV and LC-MS peak areas), and a set of chemical descriptors retrieved from the free online version of Chemicalize tool (https://chemicalize.com; accessed on 17 June 2024). As reported in Supplementary Materials (Table S1), the descriptor retrieved for each molecules were: pKa of the strongest acidic (SAG) and strongest basic (SBG) groups; distribution coefficient (LogD); neat charge (CHA); solubility (LogS); hydrophilic-lipophilic balance number (HPL); fraction of sp^3^ carbon atoms (Fsp^3^); topological polar surface area (TPSA); and polarizability (POL). The descriptors depending on pH (e.g., neat charge, logD, solubility) were calculated at pH 7.4 since the pH of the supernatants ranged between 7 and 8, consistently with literature [21].

**Figure 3:**
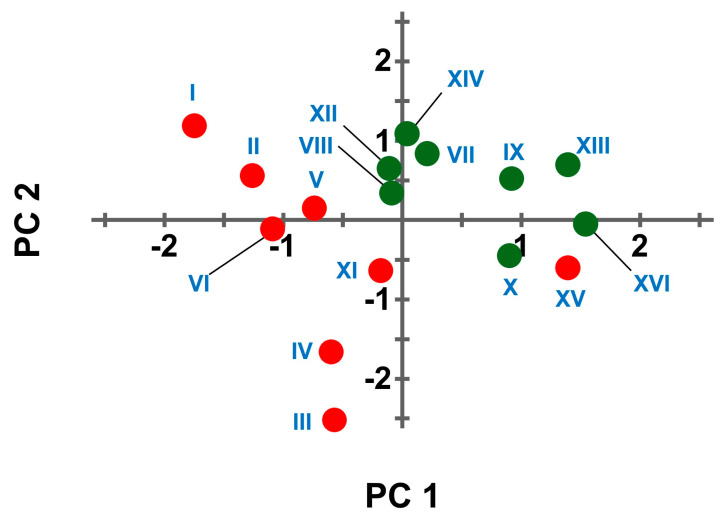
Score plot of compounds I-XVI on the first two components retrieved from Principal Component Analysis of data reported in Table 1. Compound color is based on ANOVA test (null hypothesis that concentration of compound is the same before and after precipitation of zinc hydroxide): null hypothesis rejected (red), null hypothesis accepted (green).

Principal Component Analysis (PCA) was then performed on the correlation matrix of data reported in Table S1, estimating the missing values by using an iterative imputation algorithm [24]. Figure 3 reports the score plot of the samples projected on the two principal components (i.e., PC1 and PC2) accounting for most of the data variability observed (i.e., 35.4% of total data variability for PC1 and 35.0% for PC2).

Figure 3 reports an almost complete separation of the molecules coprecipitating (i.e., red dots) and not coprecipitating (i.e., green dots) with zinc hydroxide. Such a classification was based on analysis of variance (ANOVA) with Dunnet’s multiple comparison test performed to compare the area of the chromatographic peaks before and after the precipitation with zinc hydroxide. The compounds were classified as compound coprecipitating with zinc hydroxide if ANOVA rejected with 95% confidence the null hypothesis that the area of chromatographic peaks is unaltered upon zinc hydroxide precipitation. However, PCA was performed without forcing the separation of samples according to ANOVA classification to obtain an exploratory, unsupervised analysis of the dataset.

Compounds that coprecipitate with zinc hydroxide are evenly distributed above and below the horizontal line. Therefore, PC2 does not relate with molecules coprecipitating with zinc hydroxide. On the contrary, PC1 seems to relate with molecules coprecipitating with zinc hydroxide because all of them, except bromhexine (XV), are grouped left to the vertical line. The loadings of the two main principal components of PCA are reported in Table 1 and can be easily interpreted as variables categorizing the compounds.

PC2 loadings result in a positive score for hydrophilic, soluble compounds (i.e., compound having high HLB and logS values), whereas negative scores were given to hydrophobic, polarizable compounds (i.e., compound having high POL and logD values). The loadings of PC1 suggest that positive scores are obtained for molecules with high pKa values for both the strongest acidic and the strongest basic moieties (SAG and SBG), with a high neat charge at pH 7.4 (CHA), with a low topological polar surface area (TPSA), and with a high fraction of sp^3^ carbon atoms (i.e., Fsp^3^), which has been proposed as a drug-likeness index [25]. Therefore, it looks like compound planarity and the presence of strong or medium acidic groups are associated with a higher chance of coprecipitation with zinc hydroxide.

Interestingly such a simplistic model fits also with the observed precipitation of aminoacids and proteins that have acidic groups on their scaffold. However, bromhexine (i.e., compound XV) is an exception to this general trend, since it experimentally coprecipitate with zinc hydroxide despite having the structural features of the compounds that do not coprecipitate. Therefore, there are possibly other factors affecting the interactions between zinc hydroxide and organic molecules.

Interestingly, the coprecipitation with zinc hydroxide was not only effective for removing some drugs from the supernatant but allowed also an easy and quantitative recovery of the analytes from the precipitate. Several resuspension tests were performed on a trial set composed of four molecules having different extents of coprecipitation with zinc hydroxide (i.e., compounds I; III; VI and XV). The resuspension of the precipitate was readily achievable by using aqueous solutions containing EDTA or diluted organic and inorganic acids. As an example, Table 2 reports the residual and the recovery from the precipitate using a fixed amount of zinc hydroxide (5 mM). Data were calculated upon redissolving the precipitate with 30% aqueous acetonitrile containing 0.1% formic acid.

Despite not all the compounds completely coprecipitated with zinc hydroxide, the resuspension of the precipitate allowed a complete recovery for most of the compound tested. Only for compound VI the sum of the amount detected in the supernatant (SUP) and the amount recovered from the precipitate (PRE) was less than 100%. Further experiments confirmed that complete precipitation can be achieved for all compounds by increasing the concentration of zinc hydroxide. However, the higher the concentration of zinc hydroxide, the harder the redissolution of the precipitate. Moreover, for experiments using concentration of zinc hydroxide higher than 100 mM, it was impossible to perform chromatographic analyses due to interferences with both chromatographic separation and MS detection. Therefore, the residual in the supernatant and the recovery from the precipitate were estimated from spectroscopic data performed on pure compounds.

The incomplete recovery observed for diclofenac (i.e., VI) is very likely due to a combination of lipophilicity and absence of basic residues. In fact, to redissolve zinc hydroxide precipitate aqueous solutions containing either EDTA or formic acid were tested. Such conditions are favorable for the solubility of hydrophilic compounds (e.g., acetaziolamide, I) or compounds having middle and weak basic residues (e.g., telmisartan, III; or bromhexine, XV). However, diclofenac is quite lipophilic and has no basic groups, so it is expected to be insoluble in water or diluted organic acids. The recovery of 58% reported in Table 2 was obtained because the acidified water used for the resuspension contained 50% acetonitrile. An increase of organic solvent or a change of solvent type are both easy approaches to maximize the recovery of water insoluble molecules such as VI.

### 2.2. Fractional Coprecipitation of Natural Extracts with Zinc Hydroxide

As from the results reported in the previous section, a planar scaffold with acidic groups seems conducive to the coprecipitation of small organic compounds with zinc hydroxide. The results suggest also that compounds coprecipitated with zinc hydroxide can be easily recovered from the precipitate. Hydroalcoholic extracts of natural edible materials (e.g., green tea, coffee, black pepper) were therefore used to reinforce such observations and to test the potential application of zinc hydroxide for sample fractionation.

**Figure 4:**
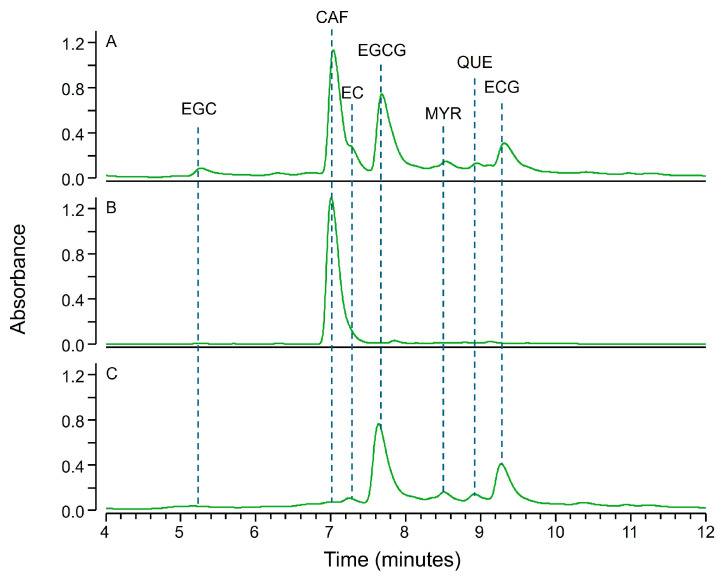
LC-UV chromatograms (260–280 nm) of a green tea hydroalcoholic extract (**A**), of its supernatant upon inducing precipitation of 100 mM zinc hydroxide (**B**) and of the solution obtained resuspending the zinc hydroxide precipitate with 30% aqueous acetonitrile containing 0.1% formic acid (**C**). Compounds identified by MS data are reported as CAF (caffeine); EGC (epigallocatechin); EC (epicatechin); EGCG (epigallocatechin gallate); MYR (myricetin); QUE (quercetin); and ECG (epicatechin gallate).

As from literature, the main constituents of green tea hydroalcoholic extracts identifiable by liquid chromatography are caffeine and a set of polyphenols classified as flavanols (e.g., catechins and catechin-gallates) or flavonols (e.g., quercetin, myricetin and kaempferol) [26,27,28]. Caffeine is not affected by zinc hydroxide precipitation, as experimentally observed on the test performed on a set of drugs (see Figure 2, compound XIV). On the contrary tea polyphenols are expected to coprecipitate with zinc hydroxide. In fact, such molecules seem to have structural features conducive to precipitation (i.e., a planar scaffold with several slightly acidic phenol groups, see Figure S2 in supplementary information). This is confirmed by the projection of the chemical descriptors of green tea polyphenols (i.e., the same retrieved for compounds I-XVI and reported in Table 1 and Table S1) on the PCA score plot reported in Figure 3. Such a projection results in PC1 and PC2 scores close to the compounds that coprecipitate with zinc hydroxide. Such a prediction was experimentally confirmed since caffeine was the only compound detectable in the supernatants of green tea hydroalcoholic extracts upon inducing the precipitation of zinc hydroxide (see Figure 4B)

Interestingly, as observed for small drugs, also green tea polyphenols were easily resuspended by using 30% aqueous acetonitrile containing 0.1% formic acid and the chromatogram of resuspended samples contained all green tea polyphenols but no trace of caffeine (see Figure 4C). The protocol was quite consistent across different natural extracts and similar results were achieved for hydroalcoholic extracts of green coffee beans. Like for green tea extracts, caffeine was the only peak detectable in the supernatant upon zinc hydroxide precipitation, and the resuspension of the precipitate allowed a complete recovery of chlorogenic acids that were the main polyphenols detectable in the extract, consistently with literature data [29,30]. This is not the first, successful example of fractional precipitation for the purification of natural polyphenols since other reagents have been tested such as supercritical fluids [31], cation exchange resins [32] or centrifugal columns packed with polyvinylpolypyrrolidone [33]. However, the protocol based on zinc hydroxide precipitation seems simpler and cheaper than such protocols.

**Figure 5:**
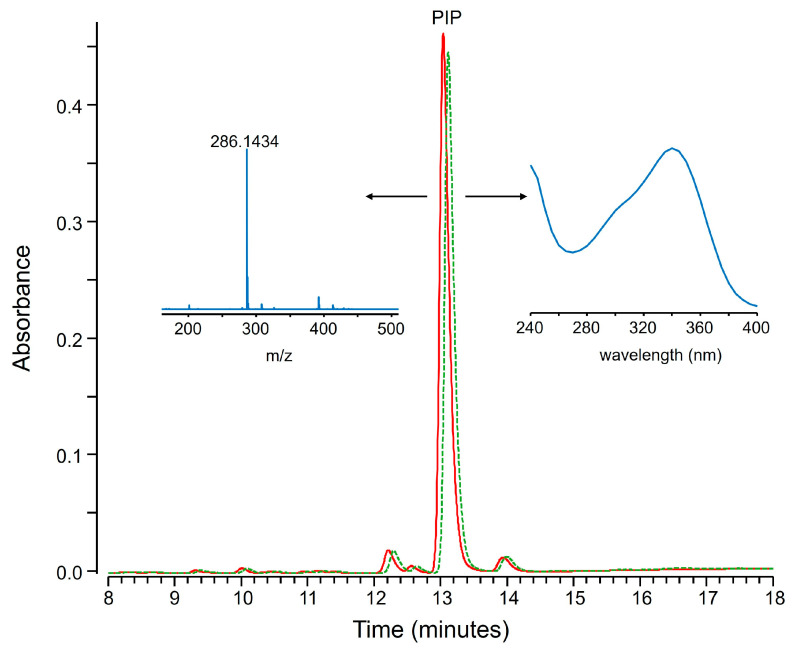
LC-UV chromatograms (320–360 nm) of a black pepper hydroalcoholic extract (green dashed line) and of its supernatant obtained inducing zinc hydroxide precipitation (solid red line). Piperine (PIP) was identified as the main peak based on UV and MS data (see inserts).

The consistency of the protocol was verified also by analyzing extracts containing natural compounds that are not expected to precipitate with zinc hydroxide. As an example, Figure 5 reports that the amount of piperine in a black pepper extract remains unaltered upon precipitation with zinc hydroxide. This is consistent with the prediction made by PCA. In fact, piperine does not have any acidic or phenolic groups (see Figure S2 in supplementary information) and gave PCA scores close to the values obtained for compound that do not coprecipitate.

The natural matrices herein reported were tested using different amount of zinc hydroxide. For instance, 5 mM zinc hydroxide ensured a complete precipitation of most green tea polyphenols, except for epicatechin and epigallocatechin. To ensure the complete precipitation of such components the concentration of zinc hydroxide must be increased up to 100 mM (see Figure 4B), which is the lowest concentration required also to quantitatively remove all chlorogenic acids from coffee extracts. However, if on one hand an excess of zinc hydroxide ensures an improved precipitation yield, on the other hand it makes the resuspension of precipitated compounds harder and sometimes an incomplete recovery from the precipitate was observed. The results suggest therefore that the amount of zinc hydroxide should be finely tuned based on sample types and based on the intended purpose. For instance, in case of green tea extract fractionation, the isolation of caffeine requires the use of 100 mM zinc hydroxide to ensure complete precipitation of all the polyphenols. On the contrary precipitation of 5 mM zinc hydroxide is more suited to obtain a mixture of green tea polyphenols since such a concentration still guarantees no precipitation of caffeine, so the precipitate contains polyphenols only and is easily redissolved.

Interestingly, it was reported that zinc and copper ions can also be used for both precipitation and resuspension of tannins [34]. The study reported an attempt to explain the mechanism involved in such a process. The pH was found to be critical parameter, and it was observed that copper seems to work better than zinc at an acidic pH where no precipitation of hydroxide is expected to occur [35]. Precipitate dissolution experiment provided data consistent with the data herein reported. Specifically, EDTA or diluted organic acids (e.g, acetic acid) were reported as efficient reagents for the recovery of coprecipitated molecules [34]. However, the precipitation of polyphenols and tannins at acidic pH resulted to be a slow process compared to the protocol herein reported, since the precipitation by metal ions required hours of incubation while zinc hydroxide precipitation requires five to ten minutes.

## 3. Materials and Methods

### 3.1. Chemicals

Water, HPLC grade (18 MΩ), was purified with a Milli-Q water system (Millipore; Milan; Italy). All the substances and chemicals reported were purchased from Sigma Aldrich (Merck Life Science, Milan, Italy). HPLC and LC-MS grade solvents were purchased from Scharlab (Scharlab Italia S.r.l, Lodi, Italy).

### 3.2. Sample Preparation

#### 3.2.1. Mixtures of Small Molecular Drugs

The study included the following compounds(see Figure S1 in supplementary information for structures): Acetazolamide (I); Piroxicam (II); Telmisartan (III); Losartan (IV); Ketoprofen (V); Diclofenac (VI); Acetaminophen (VII); Edaravone (VIII); Phenacetin (IX); Prednisolone (X); Clioquinol (XI); Hydralazine (XII); Procaine (XIII); Caffeine (XIV); Bromhexine (XV) and Fluoxetine (XVI).

For each compound a 10 mM stock solution was prepared in methanol or water and stored at −20 °C up to two weeks.

To test the effect of zinc hydroxide precipitation on small molecules three working solutions were prepared by diluting the stock solutions in 10% aqueous methanol. Mixture A was prepared with compounds I–VI, mixture B was prepared with compounds VII–XI, and mixture C was prepared with compounds XII–XVI. The working solutions used were prepared daily from the stock solutions and all compounds were diluted down to a concentration of 50 µM. For the experiments reported in Section 2.1 samples were prepared and analyzed in triplicates. Control samples were prepared as reported in Section 3.2.3.

#### 3.2.2. Extracts

The results reported in Section 2.2 were obtained by analyzing natural extracts obtained from different edible, commercially available materials. The extracts were prepared and analyzed daily. Extraction was performed in 70% aqueous ethanol for 20 min at 60 °C in an ultrasonic bath. The extracts were then filtered on first on paper filter and then on 0.45 μm OlimPeak™ syringe filters (Teknokroma, Milan, Italy). For the experiments reported in Section 2.2 all natural extracts were prepared and analyzed in triplicates. Control samples were prepared as reported in Section 3.2.3.

#### 3.2.3. Zinc Hydroxide Precipitation and Resuspension

Samples were spiked with an aqueous solution of zinc chloride and then with an equimolar solution of sodium hydroxide. The volumetric ratio of reagents was kept 2:1:1 (*v*/*v*/*v*, sample/zinc chloride/sodium hydroxide) for all samples and reagent concentration was adjusted to ensure a final concentration of zinc ranging from 10 to 100 mM. After the addition of sodium hydroxide, the samples were kept in an ice bath for 5 min and then centrifuged at 14,000 RPM for 10 min. The supernatant was then collected and the precipitate washed with five aliquots of water before being resuspended with 30% aqueous acetonitrile containing 0.1% formic acid or 100 mM disodium EDTA in water. Control samples to calculate the precipitation yield of drugs and natural extracts were prepared by adding water instead of zinc chloride and sodium hydroxide. Before chromatographic analysis, all samples were filtered by using 0.45 μm OlimPeak™ syringe filters (Teknokroma, Milan, Italy).

### 3.3. Sample Analysis

The analyses were performed by using a Surveyor HPLC connected to a LTQ Orbitrap ELITE™ mass spectrometer (MS) by using a Heated Electrospray (HESI) interface and controlled by the software Xcalibur 2.9 (Thermo Fisher Scientific, Milan, Italy).

Separations were performed at 40 °C by using a Synergi Max column (150 × 2 mm, 4 µM particle size, 80 Å pore size, Phenomenex, Milan, Italy) equipped with a 0.5 μm OPTISOLV^®^ column filter (Optimize Technologies Inc., Oregon City, OR, USA). Injections were provided by the autosampler, which was programmed to withdraw and inject 10 µL of samples in partial loop mode. Elution was provided at a flowrate of 0.2 mL/min. Water and acetonitrile containing 0.1% formic acid were used as eluents and for each sample a different linear gradient was used to ensure a sufficient peak resolution. The Surveyor diode array detector (DAD) and the MS detector were connected in series. The exit of the photodiode array detector (DAD) was connected to the divert valve of the MS instrument and the flow was diverted to the waste for the first minute of each run to avoid the contamination of the HESI interface with inorganic residues contained in the samples that can cause ionization suppression or alteration of ionization performance over time (e.g., zinc and sodium ions form zinc hydroxide precipitation). Nebulization and ionization were provided by HESI through the application of 3.5 kV capillary voltage, 30 units of sheath gas, 30 units of auxiliary gas heated at 300 °C and a capillary temperature of 200 °C.

During separation, DAD detector was scanning within a 220–400 nm wavelength range, while MS detector was scanning in positive ion mode within a 100–1000 *m*/*z* range using the orbitrap analyzer. MS data were collected at a resolution of 15,000 with default values for all the other scan parameters, except for the activation of lock mass option to use a list of the three most abundant and known background signals as reference ions for real time mass calibration. Background signals were identified based on a list reported in literature [36]. Chromatographic methods were not optimized nor specifically selected for the studies herein reported. Drugs and natural extracts were selected among a larger number of molecules/extracts by discarding compounds/extracts giving chromatographic or detection issues.

### 3.4. Data Analysis

Chromatographic peak data were processed by using the software Qual Browser (Xcalibur 2.9; Thermo Fisher Scientific, Milan, Italy). Peak assignments were done based on the match between the experimental and the expected *m*/*z* values of monoprotonated ions calculated by the function Isotope Simulation embedded in Qual Browser. Peak areas were calculated by using the default settings of the Gemini peak algorithm embedded in Qual Browser. For LC-UV data, the peak areas were extracted within a 1 nm window at the experimental maximum of absorption, whereas for LC-MS data the peaks were extracted by using the expected *m*/*z* value for the monoprotonated ions within a 20 ppm window.

For each compound, the residual amount in the supernatant after zinc hydroxide precipitation was calculated by the following formula.

$$residue=\frac{A_{s}}{A_{c}}\times 100$$

*A_s_* being the peak area of the compound in the supernatant of a sample spiked with zinc chloride and sodium hydroxide (see Section 3.2.3), while *A_c_* is the peak area of the compound in a control sample spiked with water.

The recovery from zinc hydroxide precipitate was calculated by using a similar formula.

$$recovery=\frac{A_{r}}{A_{c}}\times 100$$

*A_r_* being the peak area of the compound in the supernatant obtained from dissolution of the precipitate of zinc hydroxide with 30% aqueous acetonitrile containing 0.1% formic acid.

Principal Component Analysis (PCA) and analysis of variance (ANOVA) were performed using the most recent version of the software PAST [37]. Principal Component Analysis (PCA) was performed on the correlation matrix of data reported in Table 1. Missing values in Table 1 (e.g., pKa of strongest acidic group of drugs with no acidic groups) were estimated by using an iterative imputation algorithm [24]. ANOVA was used to classify molecules not coprecipitating or coprecipitating with zinc hydroxide. Such classification was performed with Dunnet’s multiple comparison test on the chromatographic peak areas obtained from samples treated with zinc hydroxide and control samples prepared as reported in Section 3.2.3. ANOVA was used to test the null hypothesis that zinc hydroxide treatment do not cause alteration of compound peak areas. Compounds were classified as coprecipitating when the null hypothesis was rejected with 95% confidence interval.

## 4. Conclusions

The results reported herein suggest that precipitation with zinc hydroxide can be used as an easy, convenient method for the fractional precipitation of organic compounds. This extends the range of applications of such a procedure beyond the fractional precipitation of proteins that was recently reported [21]. On small molecules, the protocol was particularly suited to separate polyphenols from alkaloids (e.g., caffeine) contained in natural extracts. However, zinc hydroxide precipitation can be potentially useful for fractional precipitation of any other mixture containing molecules with different properties. In fact, the dataset obtained by the analysis of a panel of drugs and natural extracts seems conducive to a model describing the molecules that can be precipitated by zinc hydroxide as molecules with a neat negative charge, while basic or neutral molecules seem not to be affected by zinc hydroxide precipitation. This opens some perspective for the use of zinc hydroxide precipitation for other applications. However, its applicability should be carefully evaluated case by case since also this method seems to include exceptions to the general rule, as observed for the compound bromhexine that was precipitated by zinc hydroxide despite having a structure typical of all the other molecules that resulted not affected by zinc hydroxide precipitation. However, the need for an empirical approach to find the reagent concentration suited for the intended purpose does not apply to zinc hydroxide precipitation only. Such behavior was reported also for other reagents, especially when applied to fractional precipitation of proteins [12]. The main reason for a partial unpredictability of fractional precipitation protocols is that a theoretical description of all the factors affecting the interaction of any molecule with the solvents and all the other solutes is barely impossible to obtain. However, as already reported for protein precipitation, the lack of theoretical knowledge on all the mechanisms involved in solute precipitation does not exclude a process optimization [38]. Therefore, also the fractionation of small organic molecules by zinc hydroxide precipitation is potentially applicable for controlled processes.

## Figures and Tables

**Table 1:** Loadings of the chemical descriptors on the first two components retrieved after Principal Component Analysis. Residual percentage upon zinc hydroxide precipitation (RES); pKa of the strong acidic (SAG) and strongest basic (SBG) group; distribution coefficient at pH 7.4 (LogD); neat charge at pH 7.4 (CHA); solubility at pH 7.4 (LogS); hydrophilic-lipophilic balance number (HPL); fraction of sp^3^ carbon atoms (Fsp^3^); topological polar surface area (TPSA); polarizability (POL).

	PC1	PC2
RES	0.30	0.27
SAG	0.48	0.16
SBG	0.43	−0.18
LogD	0.16	−0.48
CHA	0.50	0.08
LogS	−0.03	0.49
HLB	−0.10	0.40
Fsp^3^	0.32	−0.04
TPSA	−0.32	−0.01
POL	−0.03	−0.49

**Table 2:** Percentage of compound detectable in the supernatant obtained upon precipitation of 5 mM zinc hydroxide (SUP) or in the solution obtained dissolving the precipitate of sample A with 30% aqueous acetonitrile containing 0.1% formic acid (PRE).

Compound	SUP		PRE	
	Mean (%)	SD	Mean (%)	SD
I	4.5	0.1	99.3	0.1
III	2.2	0.1	127.8	0.7
VI	21.2	0.1	58.4	3.4
XV	15.7	0.2	85.6	0.1

## Data Availability

Dataset available on request from the authors.

## Supplementary Materials

The following supporting information can be downloaded at: https://www.mdpi.com/article/10.3390/molecules30132699/s1, Table S1. Residual percentage upon zinc hydroxide precipitation (RES) and chem-ical descriptors retrieved for all the compound tested: pKa of the strong acidic (SAG) and strongest basic (SBG) group; distribution coefficient at pH 7.4 (LogD); neat charge at pH 7.4 (CHA); solubility at pH 7.4 (LogS); hydrophilic-lipophilic balance number (HPL); fraction of sp3 carbon atoms (Fsp3); topological polar surface area (TPSA); po-larizability (POL). Figure S1. Chemical structures of the 16 drugs used for the study: Acetazolamide (I); Piroxicam (II); Telmisartan (III); Losartan (IV); Ketoprofen (V); Diclofenac (VI); Acetaminophen (VII); Edaravone (VIII); Phenacetin (IX); Prednisolone (X); Clioquinol (XI); Hydralazine (XII); Procaine (XIII); Caffeine (XIV); Bromhexine (XV) and Fluoxetine (XVI). Figure S2. Chemical structures of the main chemical components identified into the extracts of green tea, green coffee beans, and black pepper: CAF (caffeine); EGC (epigallocatechin); EC (epicatechin); EGCG (epigallocatechin gal-late); MYR (myricetin); QUE (quercetin); ECG (epicatechin gallate); and PIP (piperine).
