# Supplementary material for: Fractional Coprecipitation of Drugs and Natural Extracts with Zinc Hydroxide
Source: Molecules. 2025 Jun 23;30(13):2699. doi: 10.3390/molecules30132699 (PMC12251362; doi:10.3390/molecules30132699)

Supplementary information

Table S1. Residual percentage upon zinc hydroxide precipitation (RES) and chem-ical descriptors retrieved for all the compound tested: pKa of the strong acidic (SAG) and strongest basic (SBG) group; distribution coefficient at pH 7.4 (LogD); neat charge at pH 7.4 (CHA); solubility at pH 7.4 (LogS); hydrophilic-lipophilic balance number (HPL); fraction of sp3 carbon atoms (Fsp3); topological polar surface area (TPSA); po-larizability (POL)

CODE	NAME	RES	SAG	SBG	LogD	CHA	logS	HLB	Fsp^3	TPSA	POL
I	ACETAZOLAMIDE	3.5	6.55		-2.08	-1.44	-0.89	22.32	0.25	115.04	17.84
II	PIROXICAM	15.0	5.06	3.89	-1.39	-1.00	-0.93	20.00	0.07	99.60	32.56
III	TELMISARTAN	0.8	3.62	5.86	4.86	-0.97	-6.48	3.74	0.18	72.94	63.01
IV	LOSARTAN	12.4	5.85	3.85	3.82	-0.97	-5.01	4.31	0.27	92.51	46.46
V	KETOPROFEN	73.0	4.00		0.45	-1.00	-0.45	5.17	0.12	54.37	28.01
VI	DICLOFENAC	11.0	4.01	-1.08	1.10	-1.00	-0.90	6.08	0.07	49.33	29.03
VII	ACETAMINOPHEN	119.0	9.46		0.90	-0.01	-1.13	14.14	0.12	49.33	15.82
VIII	EDARAVONE	102.1	7.52	1.33	1.05	-0.43	-1.86	7.56	0.10	32.34	19.05
IX	PHENACETIN	105.5	14.98		1.41	0.00	-1.93	13.09	0.30	38.33	19.56
X	PREDNISOLONE	107.4	12.59		1.27	0.00	-3.46	6.59	0.71	94.83	38.04
XI	CLIOQUINOL	14.7	7.74	3.28	3.20	-0.32	-3.23	6.24	0.00	33.12	24.57
XII	HYDRALAZINE	82.3		3.90	0.75	0.00	-2.38	18.34	0.00	63.83	18.63
XIII	PROCAINE	104.7		8.96	0.31	0.97	-0.59	13.38	0.46	55.56	26.62
XIV	CAFFEINE	101.8		-1.16	-0.55	0.00	-0.44	12.10	0.37	58.44	17.86
XV	BROMHEXINE	6.3		9.23	2.59	0.99	-2.84	8.85	0.57	29.26	32.57
XVI	FLUOXETINE	89.5		9.40	2.19	0.99	-2.07	9.45	0.29	21.26	30.44

Figure S1. Chemical structures of the 16 drugs used for the study: Acetazolamide (I); Piroxicam (II); Telmisartan (III); Losartan (IV); Ketoprofen (V); Diclofenac (VI); Acetaminophen (VII); Edaravone (VIII); Phenacetin (IX); Prednisolone (X); Clioquinol (XI); Hydralazine (XII); Procaine (XIII); Caffeine (XIV); Bromhexine (XV) and Fluoxetine (XVI)

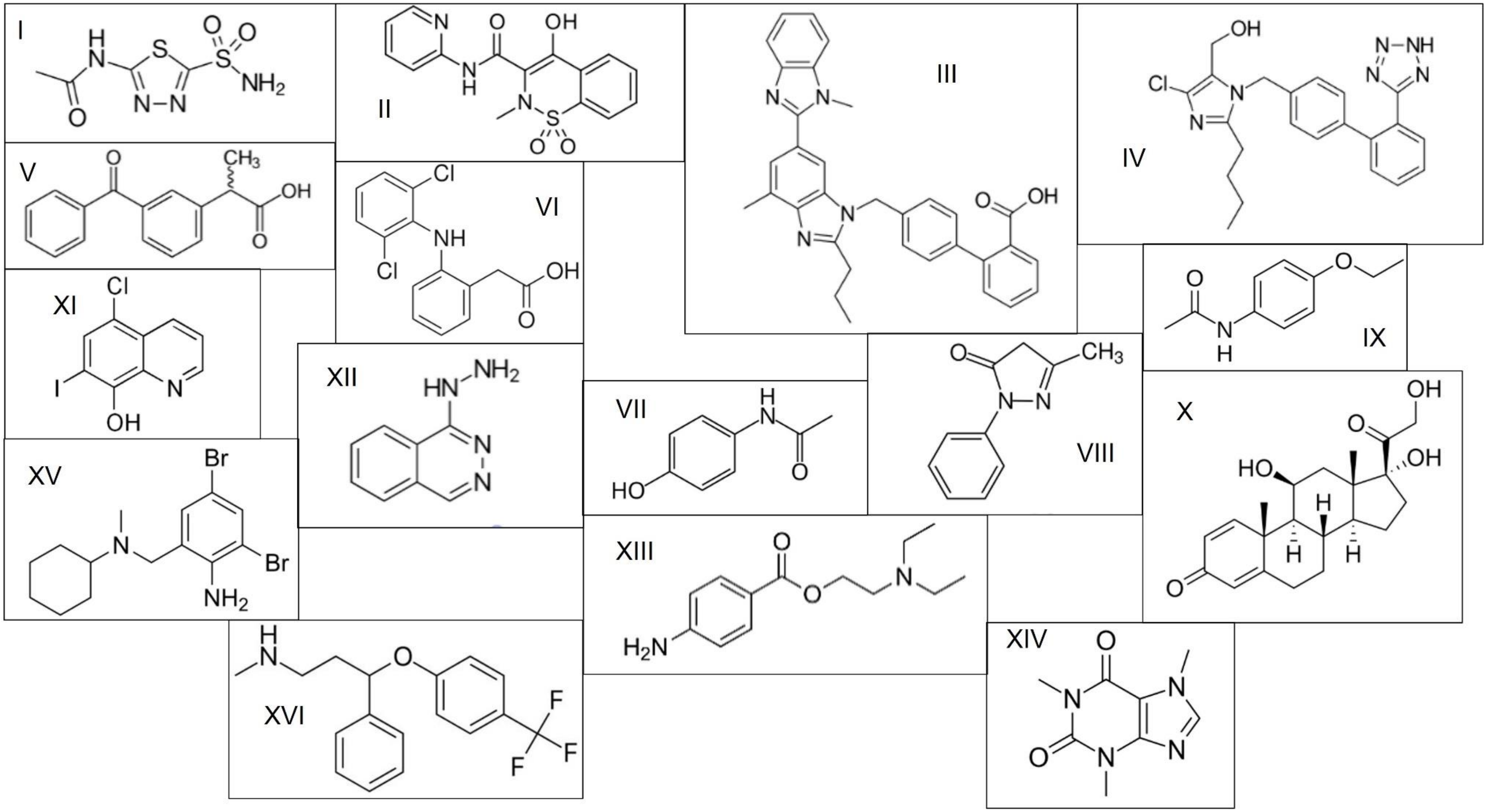

Figure S2. Chemical structures of the main chemical components identified into the extracts of green tea, green coffee beans, and black pepper: CAF (caffeine); EGC (epigallocatechin); EC (epicatechin); EGCG (epigallocatechin gal-late); MYR (myricetin); QUE (quercetin); ECG (epicatechin gallate); and PIP (piperine)

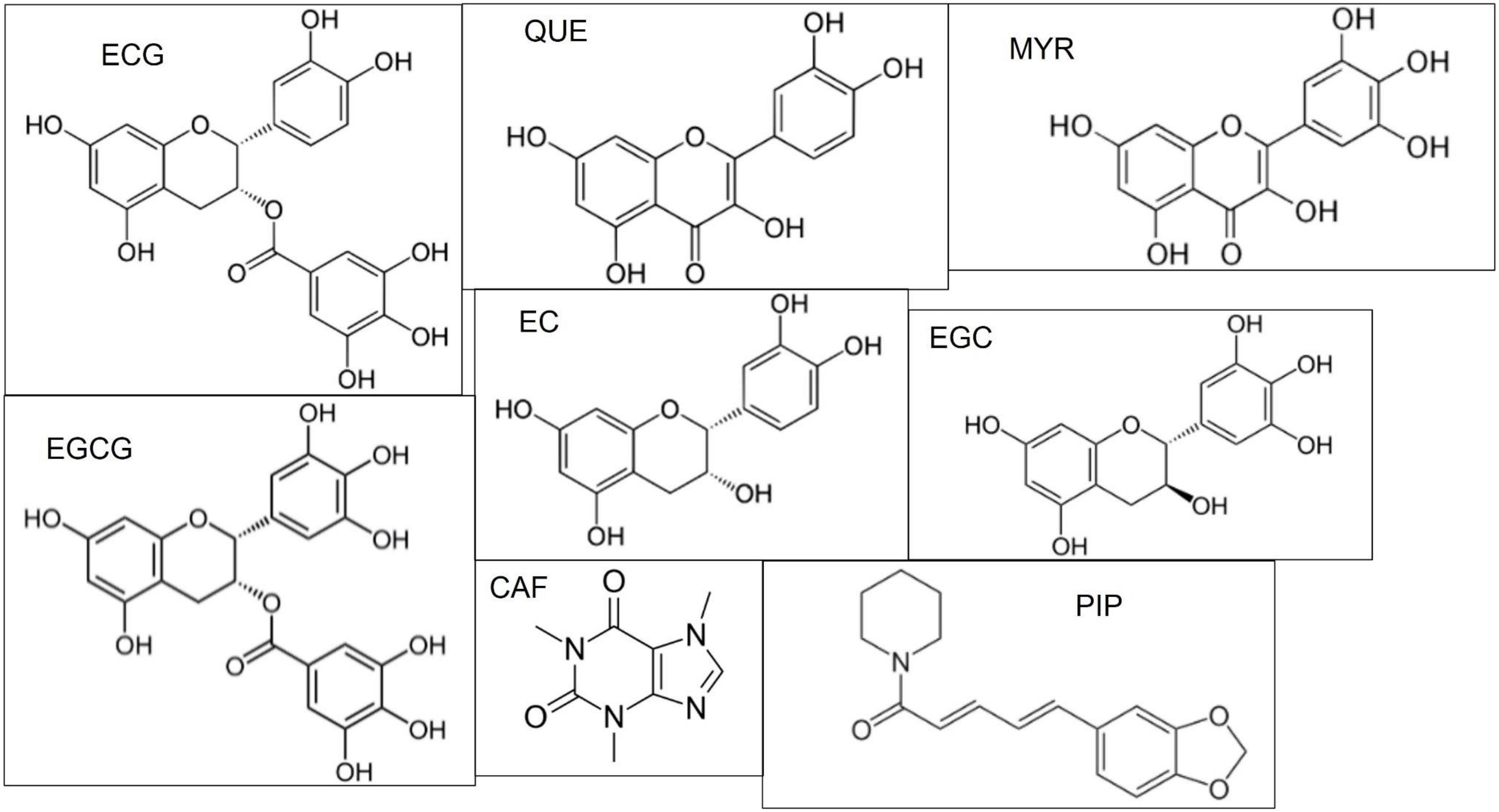

Supplement: Supplementary file 1 [file molecules-30-02699-s001.zip › molecules-3702848-supplementary.pdf]
